# Crystal clear. Engineering complexity

**DOI:** 10.1107/S2052252526006676

**Published:** 2026-07-09

**Authors:** Gautam Radhakrishna Desiraju

**Affiliations:** a Solid State and Structural Chemistry Unit Indian Institute of Science Bengaluru560012 Karnataka India; b Rishihood University, NH-44, G. T. Karnal Road, Sonepat131021, Haryana, India; c UPES University, Bidholi Campus, Via Prem Nagar, Dehradun248007, Uttarakhand, India; d Indian Institute of Technology Mandi, Kamand Campus Mandi175075 Himachal Pradesh India; Sun Yat-Sen University, China

**Keywords:** hydrogen bonds, halogen bonds, supramolecular synthons, crystal structure prediction, polymorphism, pharmaceutical cocrystals, mechanical properties of crystals, crystal engineering, Cambridge Structural Database, intermolecular interactions

## Abstract

The field of structural chemistry has been significantly reshaped by the development of crystal engineering, a subject focused on the strategic design of molecular solids with specific properties. Central to this advancement is the concept of the supramolecular synthon, the core structural module, which can be identified as a synthetic target. Such thinking enables a retrosynthetic approach to crystal design, allowing for the predictable assembly of organic solids using intermolecular interactions as tactical elements that accompany the design strategy. A major shift in our understanding of molecular architecture occurred through the validation of weak hydrogen bonds – specifically C—H⋯O interactions – as structurally and functionally relevant. Once overlooked, these interactions are now recognized for their essential roles in both small-molecule crystals and complex biological systems. The crystal engineer’s palette of interactions has been further expanded to include halogen bonding and other molecular associations involving electrophilic chalcogen, pnictogen and tetrel atoms, leading to standardized international definitions for these bonds. The culmination of these studies treats the molecular crystal not merely as an assembly of parts, but as a complex, holistic system. By framing the crystal as a supramolecular entity, the field has moved toward a predictive science that balances various intermolecular forces to engineer functional materials.

## Abstract 2

Crystal Engineering, the designed synthesis of functional molecular solids, is poised as a major branch of research in crystallography and chemistry. The centrality of this disciple is illustrated in this lead article.

## 1. Introduction

Quite apart from the fact that organic crystals were known – around the time I began my PhD studies at the University of Illinois – to exhibit chemical reactivity in ways that appeared to correlate with their internal structures (Curtin *et al.*, 1971 ▸; Paul & Curtin, 1973 ▸), there was always the question as to why these crystals took the structures they did. This was a question somewhat unrelated to the discourse between crystallography and chemistry that had been going on for the prior 50 years, indeed benefiting both subjects (Authier, 2013 ▸). Crystallography could help solve chemical questions, such as whether or not the benzene ring was planar, whether the six carbon–carbon bonds in the ring were of equal or unequal length (Lonsdale, 1928 ▸), what the structures of phthalocyanines were (Robertson, 1953 ▸), or if the Wieland–Windaus structure for steroids was correct (Bernal & Crowfoot, 1935 ▸), or even identify new and strange chemical functionalities like the β-lactam and corrin rings in natural products.

No, my early questions about molecular and crystal structures were altogether different. There was a general acceptance of the fact that crystals were close packed (Barlow, 1883 ▸). Whether they represented the closest packed possibilities in condensed matter (Hilbert, 1902 ▸) remained an unanswered question until very recently. I will come back to this later because consideration of such matters might open new vistas in a new subject, the development of which has been influenced by my research contributions, and for which I have been awarded the Ewald Prize by the International Union of Crystallography: Crystal Engineering.

## 2. Close packing

With reference to periodic arrays of organic molecules, a depiction most familiar to us, the first and major contribution to the theory of close packing of molecular crystals that I knew of when I began my PhD, was made by Kitaigorodskii (a physicist) who, by using purely geometrical considerations, showed that the mostly irregular shapes of organic molecules precluded the adoption of almost all the 230 space groups known to us (Kitaigorodskii, 1973 ▸). In reality, 67.1% of organic crystals belong to only one of three space groups (*P*1, *P*2_1_/*c*, *P*2_1_2_1_2_1_) in the three lower crystal systems, with one of them, space group No. 14, the monoclinic *P*2_1_/*c*, accounting for nearly a third of all known molecular crystal structures. Among other features that Kitaigorodskii pointed out were that the centre of inversion, the twofold screw axis and a mirror glide plane were preferred symmetry elements that facilitated close packing.

**Figure 1:**
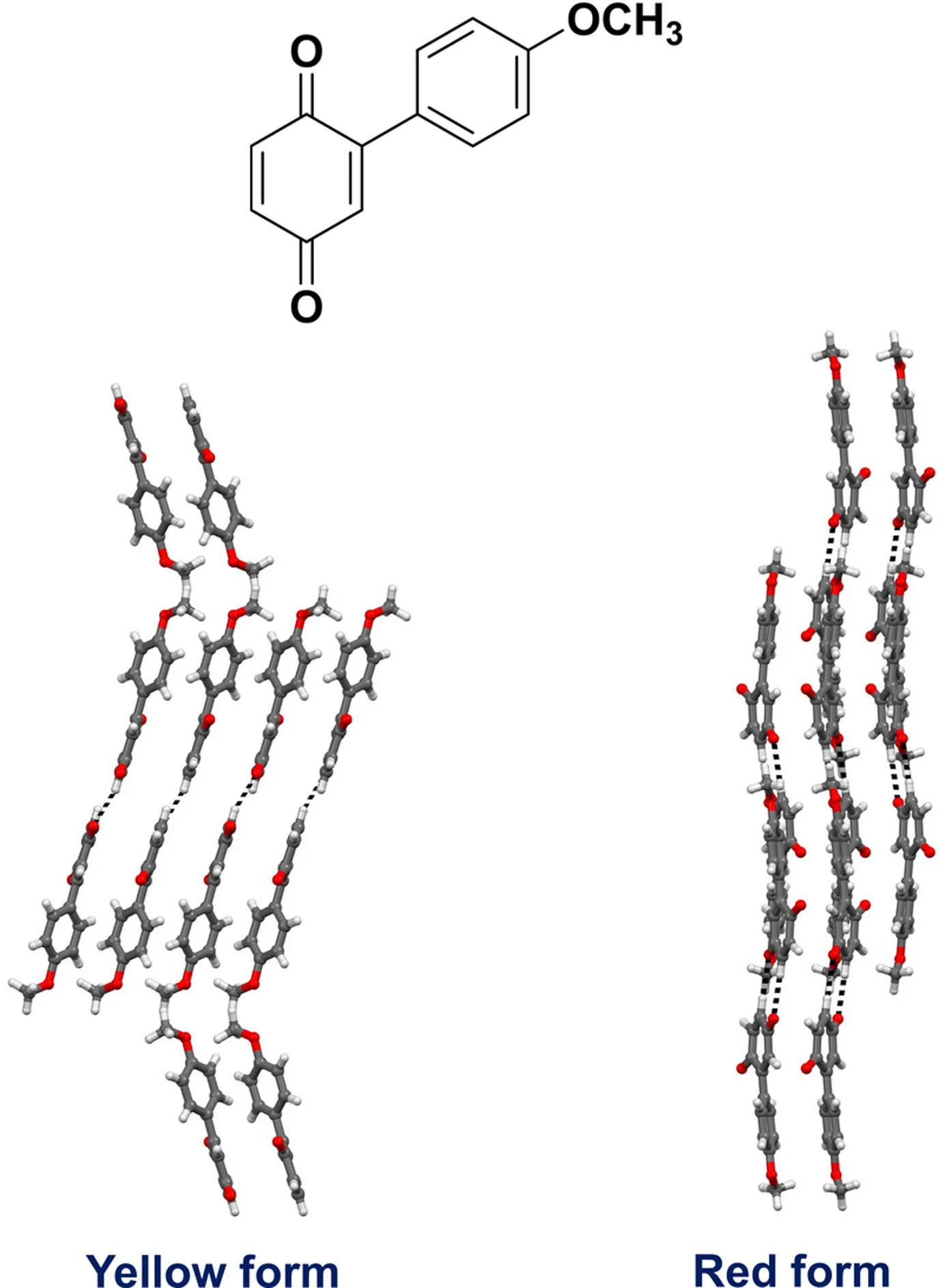
Solid state thermal rearrangement of the yellow to the red form of 4-anisyl-1,4-benzo­quinone. This study occupied half of my PhD thesis in the University of Illinois.

Another early question that remained too perplexing to me in the 1970s was why some molecules had more than one stable crystal structure. We call this phenomenon polymorphism today, and in asking this question, I had a head start over many – my PhD work dealt with molecular complexes (Pfeiffer, 1927 ▸), today’s cocrystals, of benzo­quinones and their reduction products, dihydric phenols, notably the hydro­quinones. It was known that the parent compound, quinhydrone, the 1:1 molecular complex of 1,4-benzo­quinone and 1,4-di­hydroxy­benzene (hydro­quinone), had monoclinic and triclinic forms (Sakurai, 1965 ▸, 1968 ▸). One of the quinones I made, 4-anisyl-1,4-benzo­quinone, with IUPAC name 4′-meth­oxy-[1,1′-bi­phenyl]-2,5-dione, crystallized in yellow and red forms (Fig. 1 ▸) with the yellow form transforming into the red form at a temperature 40°C below its melting point – a genuine solid state reaction (Desiraju *et al.*, 1977 ▸).

Chemistry is the study of matter in the context of its making and transformation. In the sub-domain of structural chemistry, one may move from the static geometry of crystallography with its space group and symmetry elements to a more dynamic view of molecules doing their thing: remaking themselves within the cloistered environment of the periodic crystal. Thinking about these questions in the 1980s, more in terms of crystals that did not seem to ‘obey’ well known rules of crystal packing and the attendant issue of hydrogen bonding, I felt that the exceptions were the key. A seminal contribution to the study of how hydrogen bonding influences crystal packing was made by Robertson several decades earlier (Robertson, 1953 ▸). Most accepted that hydrogen bonding was directional. Kitaigorodskii had, however, said that crystals were close packed (Kitaigorodskii, 1973 ▸). How does one reconcile these irreconcilables of solid state chemistry, directionality and close packing? How does one reconcile chemistry with crystallography?

## 3. Hydrogen bonds

Stronger varieties of the hydrogen bond, O—H⋯O=C, N—H⋯O=C, O—H⋯O—H, are characterized by intermolecular lengths and angles that occur in relatively narrow ranges (Taylor *et al.*, 1984 ▸; Murray-Rust & Glusker, 1984 ▸). Their geometries are nearly predictable. When functional groups capable of forming these strong hydrogen bonds are present, they are almost always observed. Kitaigorodskii stated, somewhat famously, that even when hydrogen bonds occurred in organic crystals, they were not inconsistent with close packing. Indeed, he was correct – the geometries of strong hydrogen bonds are fully compatible with screw axes and glide planes.

**Figure 2:**
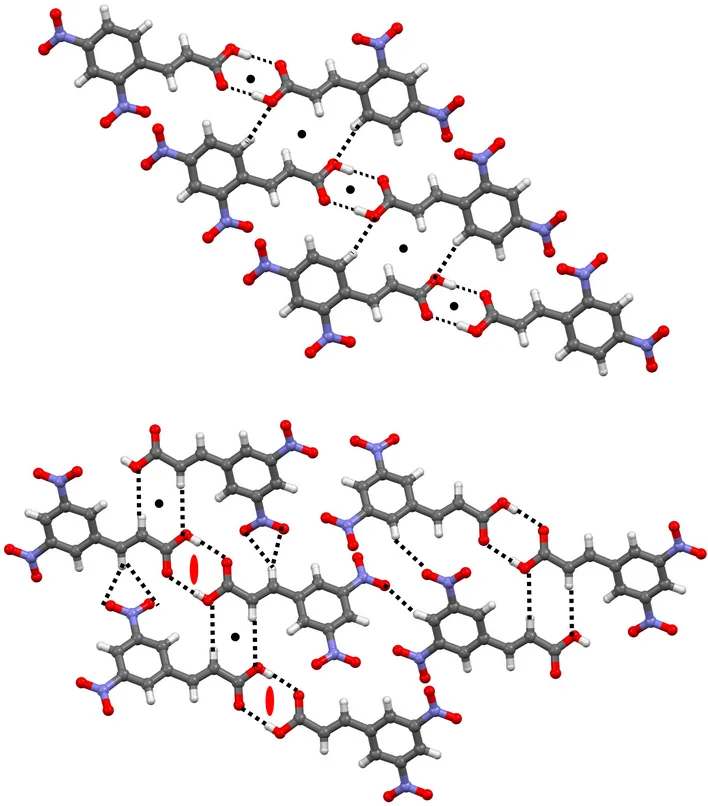
Crystal structures of 2,4-di­nitro­cinnamic acid (top) and 3,5-di­nitro­cinnamic acid (bottom) and the role of the C—H⋯O=C weak hydrogen bonds. Why do the carboxyl groups hydrogen bond around different symmetry elements in these two structures?

**Figure 3:**
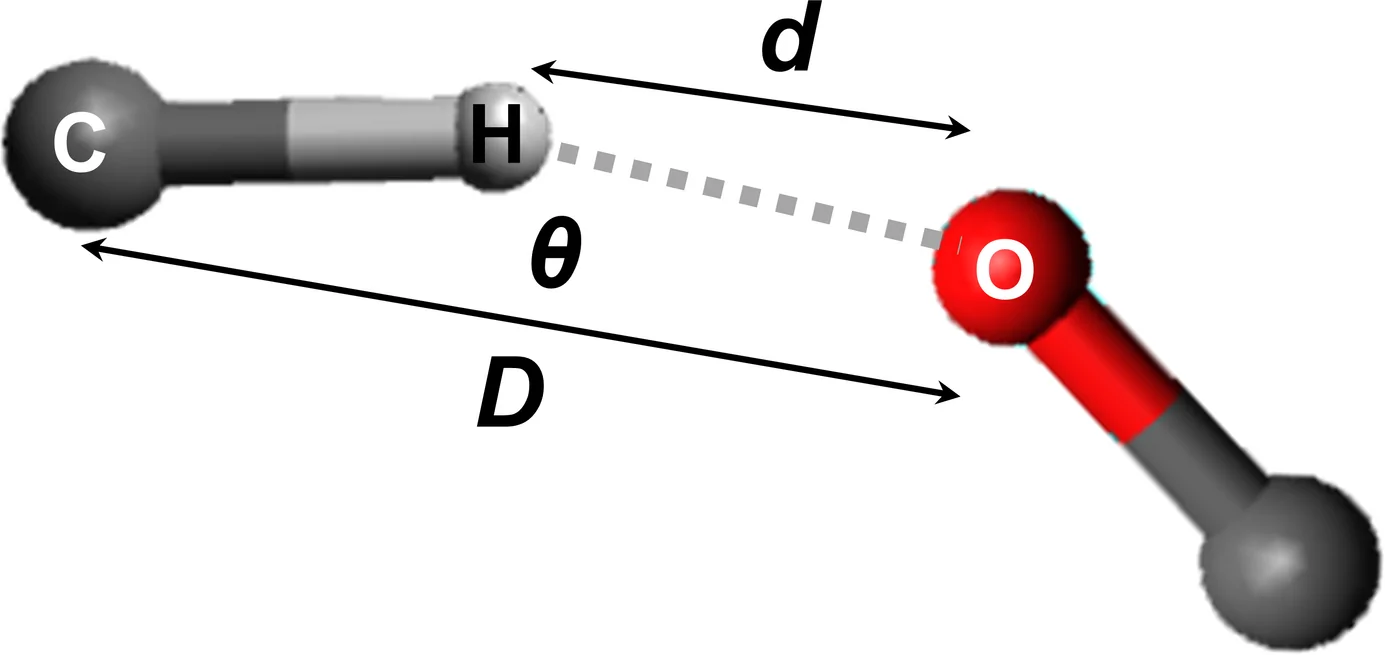
Definitions of the geometrical parameters *d*, *D* and θ for a C—H⋯O hydrogen bond. The H-atom position should be neutron-normalized for systematic analysis.

It’s when one comes to weaker interactions that the mystery really began, as far I was personally concerned. Let us look at the crystal structures of 2,4-di­nitro­cinnamic acid and 3,5-di­nitro­cinnamic acid. In the former, the carb­oxy­lic acid groups are linked with strong O—H⋯O=C hydrogen bonds across a centre of inversion as expected from close-packing theory. In the latter crystal structure, however, the acid groups bear the same topological relationship but are related by a twofold axis of symmetry (space group *C*2/*c*). Two questions arise naturally: (1) why the twofold axis at all, (2) why only in the 3,5-isomer? If one looks at these structures more closely, one observes that in the 3,5-acid, the molecules are set within a web of weaker interactions of the C—H⋯O=C type, whereas these weak interactions are not so dominant in the 2,4-acid (Fig. 2 ▸). The reasons for this are not so hard to determine. Almost every H atom in the 3,5-acid is chemically activated towards hydrogen bonding because of the particular placement of the nitro groups on the benzene ring. This is not quite the case in the 2,4-acid (Desiraju & Sharma, 1991 ▸). Examples like this and others led to the understanding of several ‘anomalous’ crystals and eventually to the establishment of the C—H⋯O interaction (Fig. 3 ▸) as a weak hydrogen bond (Desiraju & Steiner, 1999 ▸). Weak interactions (roughly in the 1–5 kcal mol^−1^ range) are the bridge connecting the ‘ideal’ geometrical crystal based on close packing to the ‘real’ crystals that are organized on chemical principles, to wit, electronegativity/hardness, acidity/basicity, reactivity/selectivity, kinetics/thermodynamics and such like.

These weak interactions are what enable the real ‘engineering’ of molecular crystals toward properties that are ‘emergent’ and have the full potential to be important in the future (Novoa, 2018 ▸; Nishio *et al.*, 1998 ▸; Bakhmutov, 2008 ▸). Strong interactions allow for predictability of crystal structures, but adapting them to properties that are of more utility in the future, solid state reactions, photocatalysis, AI-based properties such as the design of lower-dimensional crystals, soft matter, aperiodic crystals and so on, becomes increasingly challenging (Ohashi, 2014 ▸). By using weaker interactions in the design process, one introduces the elements of property design into the structure design itself. Traditionally, these two stages were taken separately and sequentially – today they could be taken together for mutual advantage.

In my well known 1989 book (Desiraju, 1989 ▸), which several have opined was a new beginning for crystal engineering, I defined it as ‘the understanding of intermolecular interactions in the context of crystal packing and utilization of such understanding in the design of new solids with desired physical and chemical properties’. Three elements of crystal engineering – structure, design and property – are involved here. My work over the last at least 35 years has shown that these three elements are not mutually exclusive. Indeed, the converse is true. The consideration of these three phases of development *together* is what takes one from the realm of the simple to that of the complex.

A great deal of my research time in the 1990s had to do with this most baffling and enigmatic of intermolecular interactions, namely the ubiquitous ‘hydrogen bond’. Is it a bond? Is it merely an ’intermolecular interaction’? Does a bond differ from an interaction? Even assuming that ‘bond’ is a chemical idea, the reader will note that all bonds are interactions, howsoever these interactions are defined, whether by chemists or crystallographers. Most structural chemists agreed that it has electrophilic character because of polarization of the *X*—H covalent bond so that the H atom acquires a positive charge with respect to the *X* atom, so that it is attracted to the acceptor atom *Y* forming an intermolecular conglomerate *X*—H⋯*Y*—*Z* which one could call a ‘hydrogen bond’ (Gilli & Gilli, 2009 ▸). This hydrogen bond, in all its many variations, is truly the master key of molecular recognition (Jeffrey, 1997 ▸; Jeffrey & Saenger, 1991 ▸). The H atom with its small size and single valence electron is the pivot of a four-atom ensemble with a variety of donor atoms *X*, that include notably, and in addition to O, N, C (Pedireddi & Desiraju, 1992 ▸), S, even metals (Braga *et al.*, 1998 ▸; Aullón *et al.*, 1998 ▸) and metalloids (Resnati *et al.*, 2024 ▸). As for *Y*, there are a variety of nucleophilic atoms or groups of atoms that are perfectly good acceptors. These include, in addition to O, N, and halogen, species such as multiple bonds, aromatic rings and metal atoms.

We note that metal atoms, under suitable conditions, may act as donors or acceptors. There are practically an endless number of hydrogen bonds, this term being taken in its wider context, in the toolkit of the crystal engineer; many, myself included, have made ample use of this interaction, an essential part of the methodology of crystal engineering. Much of this culminated in my 1999 book, written with Thomas Steiner, where we defined the weak hydrogen bond as ‘an interaction *X*—H⋯*Y*—*Z* wherein a hydrogen atom forms a bond between two structural fragments *X*—H and *Y*—*Z*, in which one, or even both, of the atoms *X* and *Y* are of moderate to low electronegativity’ (Desiraju & Steiner, 1999 ▸). Ten years on, I was part of an IUPAC committee that redefined the hydrogen bond as ‘an attractive interaction between a hydrogen atom from a molecule or a molecular fragment *X*H in which *X* is more electronegative than H, and an atom or group of atoms in the same or a different molecule, in which there is evidence of bond formation’ (Desiraju, 2011 ▸). The reader will note the use of the word ‘bond’ in the expanded definition. This is a ‘chemistry’ word in contrast to ‘interaction’, which may be a ‘crystallography’ word. Words in science are strange things because they are the routes through which scientists seek attribution, adherence and appropriation. Debates over words and nomenclature are almost always a feature of evolving subjects. I have been personally involved in many of these discussions as they pertained to cocrystals, weak and strong interactions and supramolecular synthons, the other big exercise in which I played a major role, having defined the term myself.

**Figure 4:**
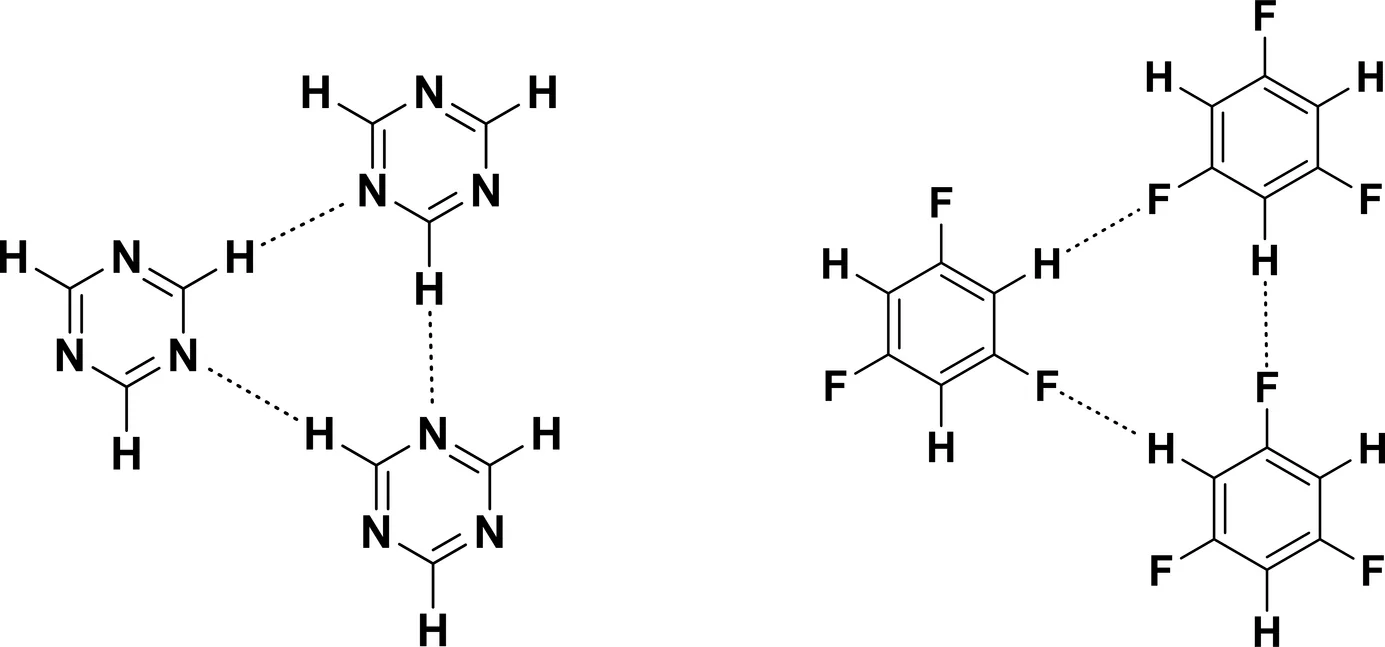
Interaction mimicry. The synthons are the ‘same’, and so the interactions are the ‘same’. If the C—H⋯N interaction is taken as a hydrogen bond, then so should the C—H⋯F—C interaction.

Let me say that when one goes in there to disprove an interaction, one picks the strongest example that one’s scientific competitor can advance. When one attempts to prove one’s own interaction, one picks the weakest example in one’s armoury. Such was the case in my many battles concerning C—H⋯F—C hydrogen bonds during the years 2000–2010 (Fig. 4 ▸), which involved the so-called ‘organic fluorine’, a notoriously hard (in the hard/soft sense) hydrogen-bond acceptor and a weak C—H donor (Thalladi *et al.*, 1998 ▸; Thakur, Kirchner *et al.*, 2010 ▸). I am reasonably pleased that the structural community has accepted this interaction as belonging in the domain of weak hydrogen bonds.

Playing with words, I wrote an article in 2002 where I advocated for a return to the older German word ‘hydrogen bridge’ or *Wasserstoffbrücke* (Desiraju, 2002 ▸). The ‘bridge’ word was a linguistic way of avoiding what was not even a chemical problem and it more accurately described certain essential facts of the new subject of supramolecular chemistry that emerged out of the study of host–guest molecular complexes (Cram, 2007 ▸), and which had a major impact on the way I defined crystal engineering as a form of organic synthesis in 1995 in my now well known article on supramolecular synthons (Desiraju, 1995 ▸). Crystal engineering, I argued, was an integral part of chemistry because it is a form of synthesis and chemistry is nothing but synthesis and all the attendant disciplines that assist the overall synthetic effect, both in terms of methodology, to which I have already referred when I spoke about hydrogen bonds, and in terms of strategy, the grand chessboard itself.

## 4. Supramolecular synthons

**Figure 5:**
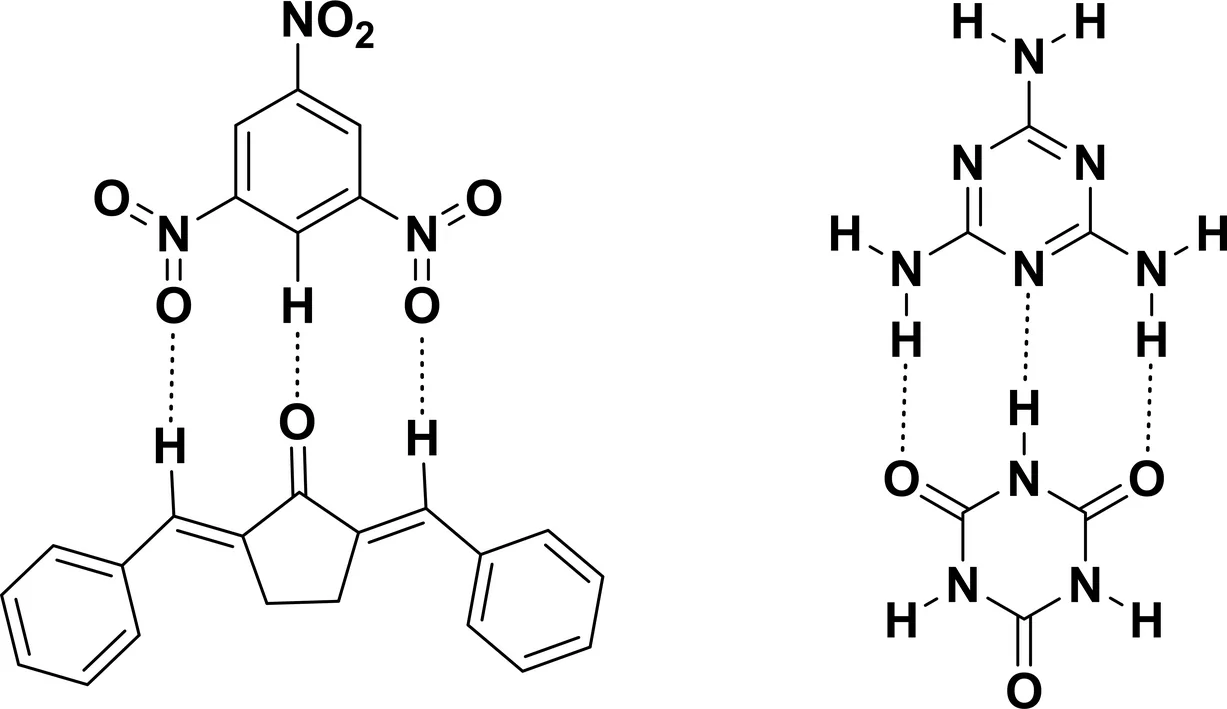
Interaction mimicry. The C—H⋯O pattern on the left is equivalent to the N—H⋯O and N—H⋯N pattern on the right. These patterns are equivalent supramolecular synthons.

My studies on weaker hydrogen bonds took me implicitly to crystal structures where the arrangement of these bonds was topologically reminiscent of those in crystal structures containing stronger hydrogen bonds (Fig. 5 ▸) (Biradha *et al.*, 1993 ▸). This phenomenon of ‘interaction mimicry’ had been observed previously in molecular complexes of 5,5-di­ethyl­barbituric acid with acetanilide and urea (Berkovitch-Yellin & Leiserowitz, 1984 ▸; Thakur, Azim *et al.*, 2010 ▸), and also elsewhere. I noticed more examples where C—H⋯O bonds formed by molecules with activated C—H groups bore strikingly similar relationships with patterns seen in molecular complexes of say, melamine and cyanuric acid, a popular compound in the early 1990s among those interested in a subject that was called ‘molecular recognition’, in itself an offshoot of a broader discipline called ‘supramolecular chemistry’ largely initiated by Lehn and concerned with associations between molecules to give aggregates whose properties were more than the sum of the properties of the individual molecules themselves (Lehn, 1995 ▸).

Initially, these ‘supramolecular’ aggregates took the form of host–guest complexes, basically zero-dimensional species (Cram, 2007 ▸). With the recognition that the three-dimensional molecular crystal with its periodic array of molecules is a supramolecular entity, it was but a short step for me to start thinking of a molecular crystal as a synthetic target; I was being increasingly drawn into the chemical mainstream.

**Figure 6:**
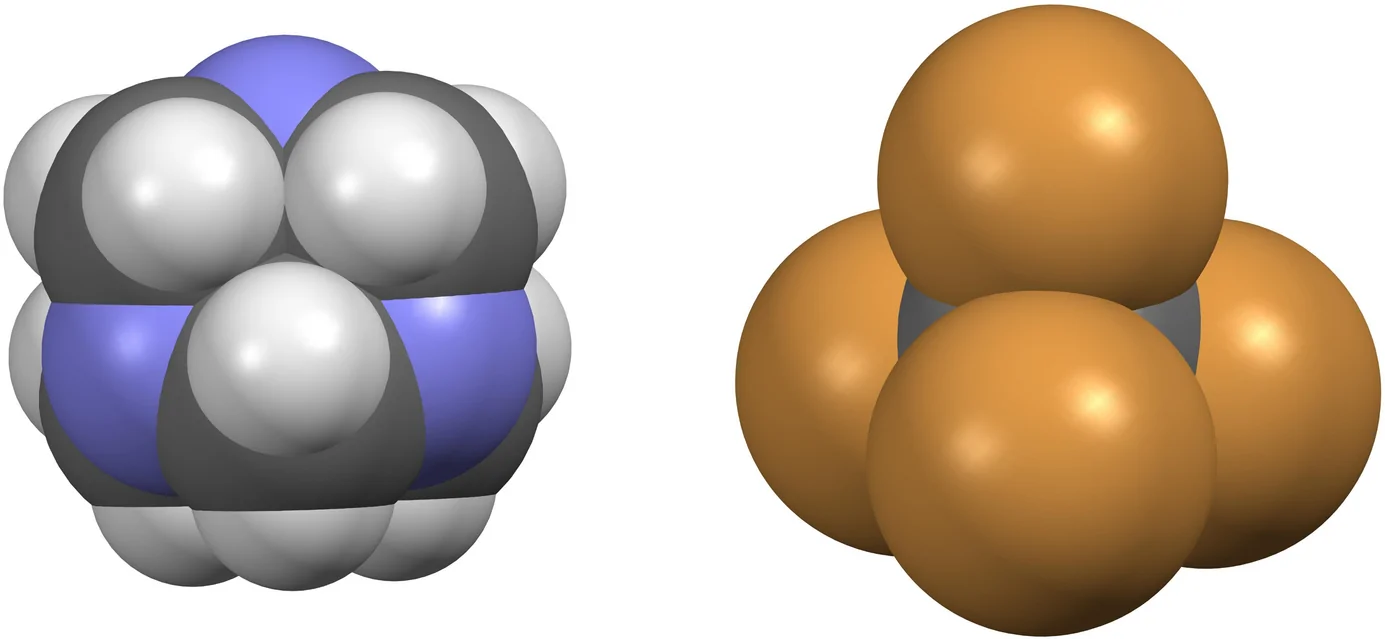
Urotropin and carbon tetrabromide are of the same shape and size. The one can substitute for the other in cocrystals with 1,3,5,7-tetra­bromo­adamantane.

Consider for example, the molecules 1,3,5,7-tetra­bromo­adamantane and hexa­methyl­enetetramine (or urotropin) that cocrystallize to form a 1:2 complex where the same tetrahedral molecular symmetry of both molecules and the linear nature of the polarization induced N⋯Br interaction (we now would call it a halogen bond) permits the adoption of a cubic space group. The two urotropin molecules have different site symmetries, a feature that I have used repeatedly in design strategies in subsequent years: divide and rule. One of the molecules is halogen bonded as described above; the other is disordered and is situated at the centre of the unit cell. This second molecule can be replaced by CBr_4_, a molecule of roughly the same size and shape (Fig. 6 ▸), so that one obtains a stoichiometric ternary aggregate (Reddy *et al.*, 1994 ▸). We would call it a ternary cocrystal today. In this example, it is the supramolecular structure that becomes the synthetic target; the N⋯Br interaction when employed with symmetrical molecules leads to a tetrahedral network.

It was an equally short step at that stage to define the ‘supramolecular synthon’ as a ‘structural unit within supermolecules which can be formed or assembled by known or conceivable interactions’. The reader will notice the words ‘synthetic’ and ‘intermolecular’, as this definition bears a close relationship to its molecular equivalent, the ‘molecular synthon’, which was proposed by Corey in 1967 when he laid down the principles of retrosynthesis for complex molecular targets (Corey, 1967 ▸). The process of disconnection of a molecular structure into fragments that were easily accessed through known and conceived reactions (Warren, 2002 ▸) may be followed more or less directly in a disconnection of a crystal structure, notably in the supramolecular regions, so that a structure, when defined as a ribbon, strip, layer or in its three-dimensional entirety, may be accessed through logically derived molecular components.

**Figure 7:**
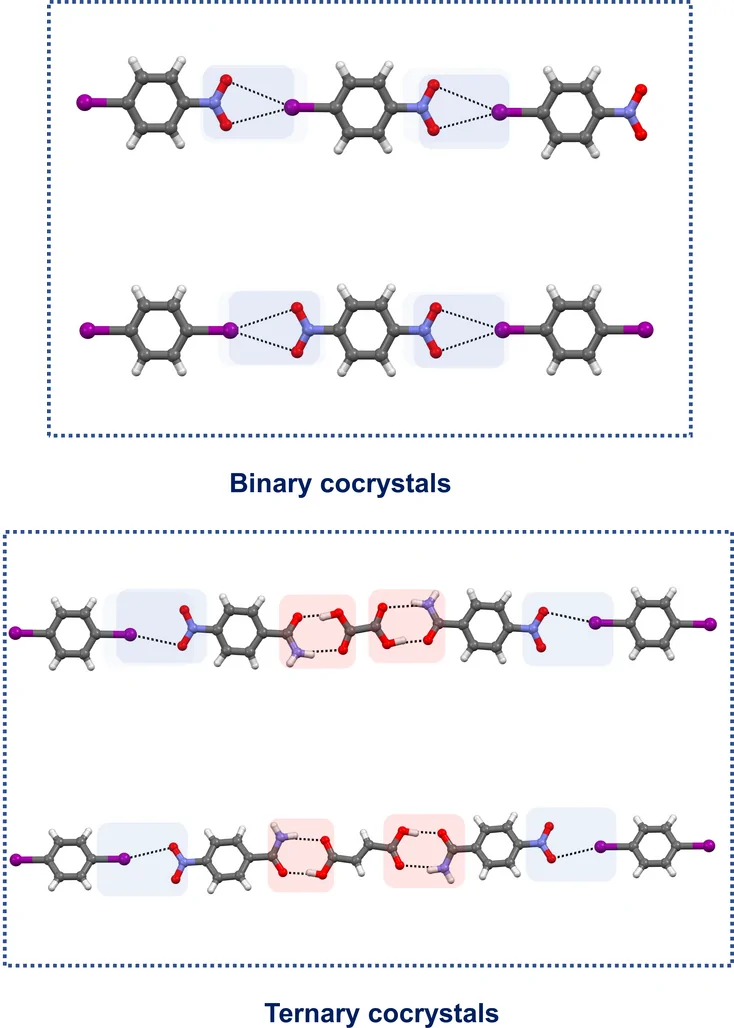
Crystal Engineering. Top: different molecular structures can have the same supramolecular structure. Bottom: homologation from a binary to a ternary cocrystal is possible with alternative insulated synthons. Colour coding: blue, halogen bonding; pink, hydrogen bonding.

Accordingly, the reader will note that the crystal structures of 4-iodo­nitro­benzene and the 1:1 molecular complex of 1,4-di­iodo­benzene and 1,4-di­nitro­benzene are equivalent (Fig. 7 ▸). The two structures are, supramolecularly speaking, the same and take the topological form of a linear tape of 1,4-disubstituted phenyl rings connected with iodo⋯nitro synthons (Thalladi *et al.*, 1996 ▸).

The synthon is the heart or critical core of an organic crystal structure (Desiraju, 2000 ▸). The arrangement of synthons is what uniquely defines a crystal structure (Nangia & Desiraju, 1998 ▸). The synthon is what is obtained in the retrosynthesis of a solid state supramolecular target. Synthons may be of varying sizes; the smaller the synthon and the more information dense it is, the more useful it will be in crystal engineering. *The synthon is the strategic component of the synthetic effort; the intermolecular interactions that constitute the synthon represent its methodological component.* If I take poetic licence and liken synthesis, and this includes chemical synthesis, to war, it is composed of strategy and tactics (Tzu, 1963 ▸). The balance between these two constitutes the ‘art’ of chemical synthesis while the ‘robustness’ of the supramolecular synthons, in other words the spatial reproducibility and consistency of their constituent intermolecular interactions, constitutes the ‘science’.

One may proceed from the linear tapes in the crystal structures of the iodo­nitro­benzenes described above to binary and ternary cocrystals with alternating halogen-bonded (Metrangolo *et al.*, 2005 ▸; Mukherjee, Tothadi & Desiraju, 2014 ▸) and hydrogen-bonded supramolecular synthons (Tothadi & Desiraju, 2013 ▸) (Fig. 7 ▸). It is of note that when halogen- or nitro-substituted acids and amides are cocrystallized, the hydrogen-bonded functionalities do not ‘recognize’ the nitro groups; the halogen-bonded I⋯NO_2_ synthons (Thaimattam *et al.*, 2001 ▸) are ‘well insulated’. Synthon insulation becomes an important part of higher cocrystal design, and the fact that various synthons contribute differently to the overall crystal stabilization is vital to the formation of multicomponent solids (Aakeröy *et al.*, 2001 ▸; Jain *et al.*, 2021 ▸). If all interactions, and *inter alia*, the synthons that result from them, are of equal energetic significance, what might have been obtained could have been intractable mixtures, basically conglomerates, of single and various multicomponent solids. Typically, if *A*, *B* and *C* were taken together for crystallization and if the energies of *AA*, *BB*, *CC*, *AB*, *AC* and *BC* were all comparable, there would be a whole host of solids obtained, single, two and rarely, if at all, the desired three-component solids *ABC*. In practice, across a large number of experimental runs using well chosen compounds *A*, *B* and *C*, we usually obtained pure, stoichiometric ternary solids *ABC*.

**Figure 8:**
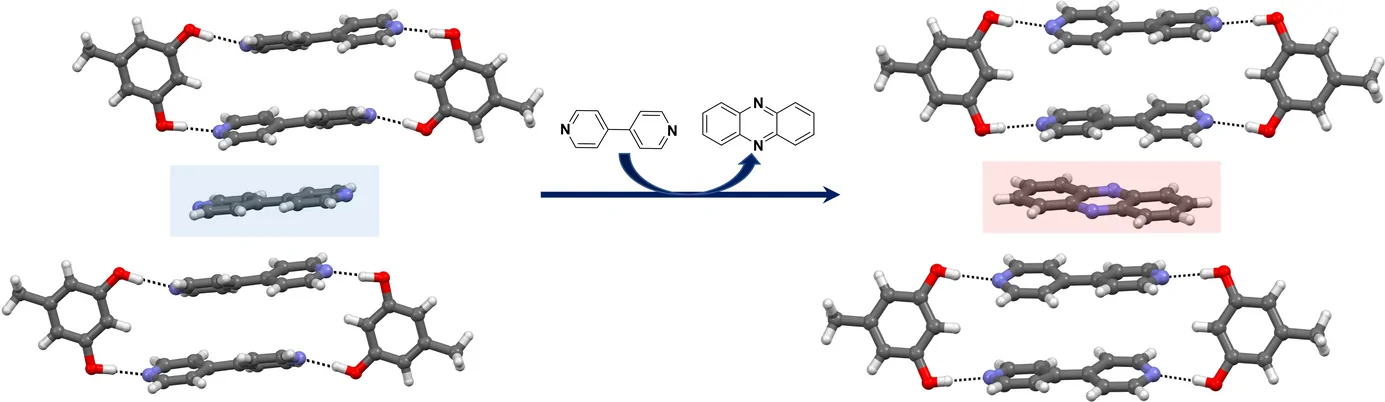
Homologation from a binary to a ternary cocrystal illustrating volume replacement.

In a variation of these themes, a combination of source synthons modified with shape-size mimicry of one of its molecular components can result in higher stoichiometric multicomponent crystals. In a representative example, 2- and 5-methyl resorcinol form cocrystals with 4,4′-bi­pyridine in which some of the latter molecules are loosely bound. These molecules that are held more loosely can be replaced with other molecules of a similar shape and size. This allows the crystal engineer to access a more general protocol for synthesis (Fig. 8 ▸) (Tothadi *et al.*, 2011 ▸).

Beyond a point, it is unnecessary and impractical to distinguish between molecules and synthons. Both make up the holistic crystal. In an intriguing example, a geminal alkynol, *trans*-1,5-di­chloro-9,10-diethynyl-9,10-di­hydro­anthra­cene-9,10-diol, a molecule that lies on a molecular inversion centre, adopts a centrosymmetric space group and the molecular inversion centre, rather than occupying the crystal centre of symmetry, as Kitaigorodskii might have had it, sits on a general position (Banerjee *et al.*, 2003 ▸). The crystal inversion centre is occupied by an inversion-symmetrical Cl_4_ supramolecular synthon! Looking back at this structure, one realizes that its unusual features owe their origin to the fact that the hydrogen-bond donors and acceptors are in sterically hindered environments in the molecular sense – the hydrogen bonds they manage to form are also unusual, and the Cl_4_ synthon assumes a greater importance in the overall packing.

**Figure 9:**
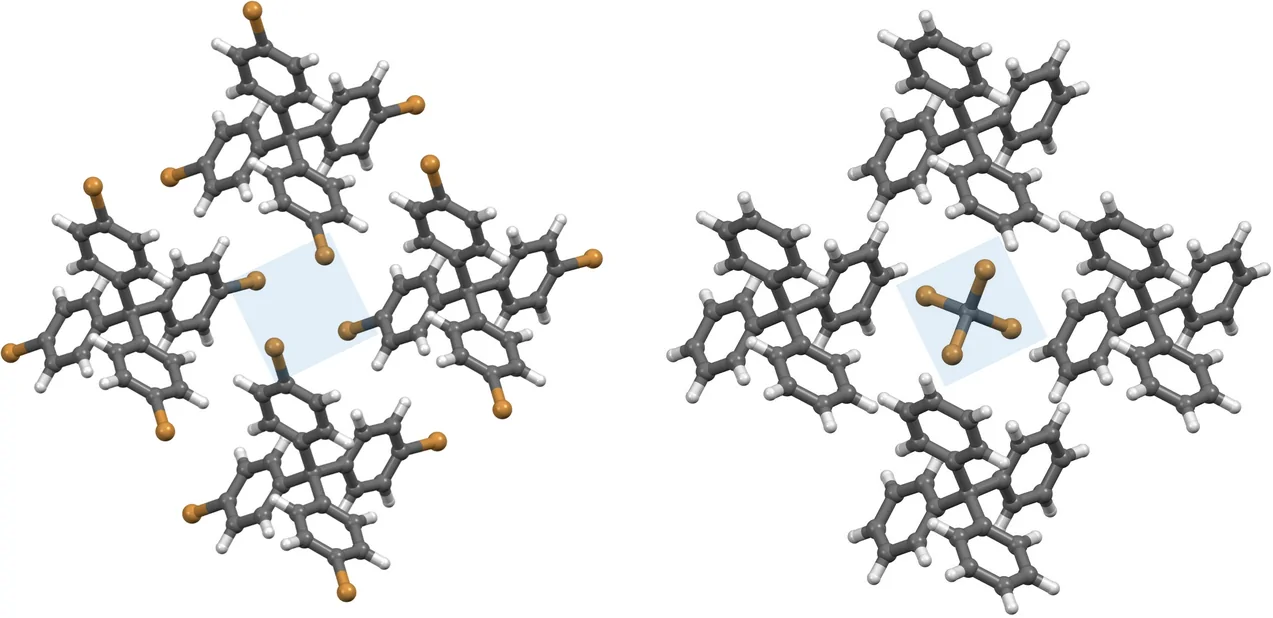
Molecules and synthons of tetra(4-bromo­phenyl)­methane and the 1:1 cocrystal of tetra­phenyl­methane and CBr_4_.

Similarly, the crystal structures of tetra(4-bromo­phenyl)­methane and the 1:1 cocrystal of tetra­phenyl­methane and CBr_4_ are almost the same (Fig. 9 ▸). For me, the molecular synthon CBr_4_ and the supramolecular synthon CBr_4_ are equivalent in terms of crystal packing (Reddy *et al.*, 1996 ▸).

Interaction hierarchy and synthon insulation may be used for increasing levels of supramolecular homologation. We were particularly fortunate in that we were able to extend the synthesis of stoichiometric quaternary cocrystals to systems of N-heterocycles, polyhydric phenols and polynuclear aromatic hydro­carbons using hydrogen bonds, halogen bonds and π⋯π stacking interactions (Mir *et al.*, 2016 ▸; Dubey *et al.*, 2016 ▸; Mir *et al.*, 2019 ▸; Paul & Desiraju, 2019 ▸). These baskets of higher cocrystals were seen to have a greater variety of intermolecular interactions.

**Figure 10:**
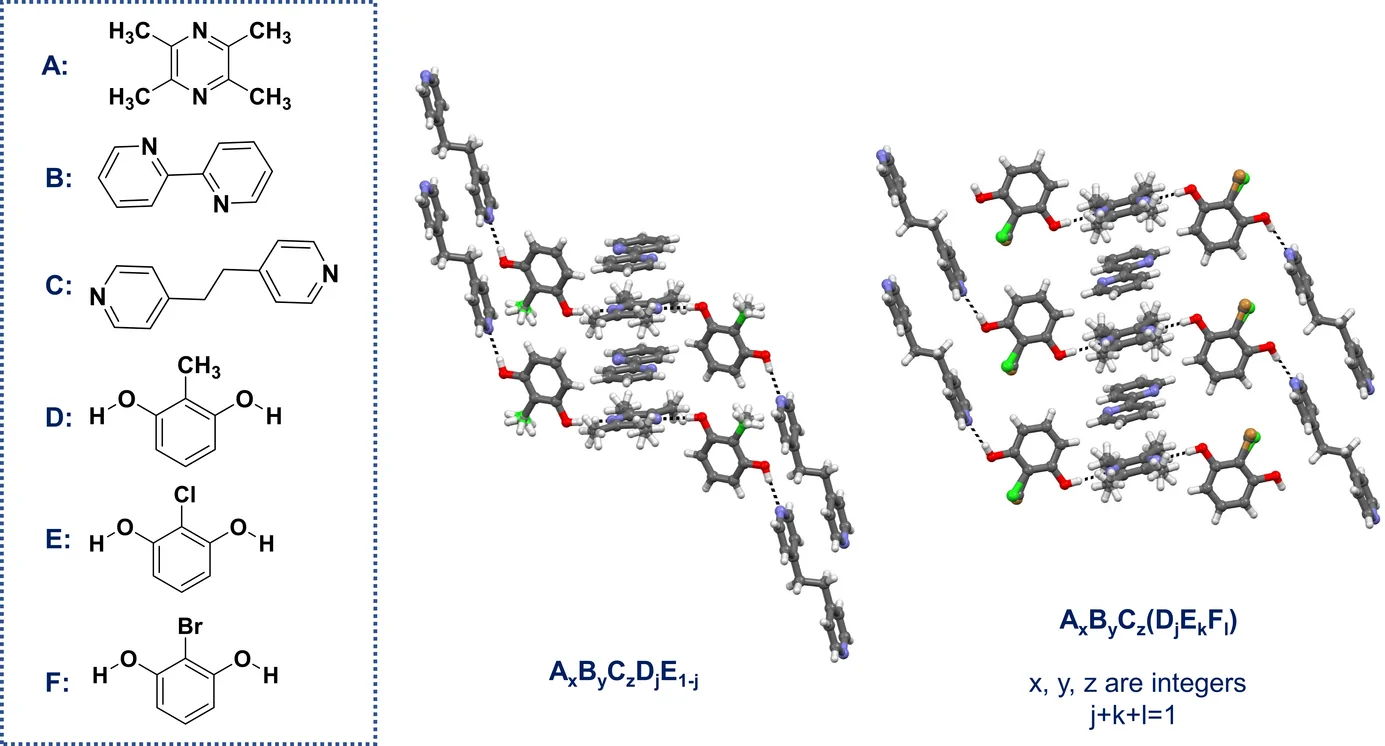
A six-component molecular crystal. Molecules *A*, *B* and *C* sit on ordered sites. *D*, *E* and *F* are substitutionally disordered in the manner of solid solutions.

The carefully chosen strengths of these interactions constitute the core of the synthetic exercise. If solid solution formation is also invoked, the synthetic exercise may be extended to higher homologation to five- and six-component molecular crystals *A*_*x*_*B*_*y*_*C*_*z*_*D*_*j*_*E*_1−*j*_ and *A*_*x*_*B*_*y*_*C*_*z*_(*D*_*j*_*E*_*k*_*F*_*l*_) where *x*, *y*, *z* are integers and when *j* + *k* + *l* = 1 for the six-component crystal (Fig. 10 ▸) (Paul *et al.*, 2018 ▸). A key ingredient of supramolecular homologation in these cocrystals is the preferential substitution of a molecule that occupies one of two crystallographically distinct environments at only one of the two positions. Whether or not a stoichiometric five-component will be realisable in the future is an open question and hinges on whether four intermolecular interactions of sufficiently different strengths can be identified; additionally, their directional preferences should be compatible with the overall requirements of close packing. This is a tall order, and the exercise poses a synthetic challenge of the highest magnitude, the Mount Everest of supramolecular solid state synthesis.

## 5. Pharmaceutical cocrystals

The synthesis of multicomponent molecular crystals is not only of high intellectual appeal but also of utter practical applicability. The utility of cocrystals, wherein at least one component is an active pharmaceutical ingredient, or API, in other words a drug, is of direct interest to the pharmaceutical industry. This has resulted in pharmaceutical cocrystals taking the form of marketable drugs, several of which are on the market today, nearly 20 years after the introduction of the term ‘pharmaceutical cocrystal’ in the open scientific literature (Almarsson & Zaworotko, 2004 ▸). This highly compressed time scale of events was only possible because developments in pure and applied pharmaceutical sciences took place synchronously rather than sequentially. We are almost at the stage where cocrystal formation of a new innovator API becomes a viable part in the design cycle itself, rather than a fully designed and patented molecule being handed over to solid state chemists for cocrystal development (Nangia & Desiraju, 2022 ▸). There is a real reason for this; many of the molecules developed as APIs using software and AI approaches tend to bring up lipophilic species with poor water solubility, the so-called class 2 and class 4 drugs if one were to use the Biopharmaceutics Classification System (BCS) nomenclature.

Two main problems associated with the poor bioavailability of newly developed drugs are their limited water solubility and limited permeability, which prevent them from crossing the water–lipid membrane barrier in the small intestine. As might have been expected, the molecular characteristics associated with good solubility and good permeability run counter to each other. Molecules that contain ionizable, polar or hydrogen-bonding functional groups will increase water solubility, but these are exactly the features that depress the permeability, which requires more lipid-like molecules with appropriate functional groups. The possibility of exploring molecules capable of exhibiting weaker types of hydrogen bonding (C—H⋯O, C—H⋯N, possibly even C—H⋯F—C) would help in such efforts (Mukherjee & Desiraju, 2014 ▸).

The main strategy in this exercise is therefore geared towards designing a drug that optimizes both water solubility and lipid permeability. Accordingly, one seeks a situation where the drug molecule is persuaded to form a two-component crystal with a molecule known as the ‘coformer’ through a set of intermolecular interactions so that they constitute what we now call a ‘heterosynthon’ (Almarsson & Zaworotko, 2004 ▸). This term was coined supposedly to distinguish these synthons from what could presumably be referred to as ‘homosynthons’, although my 1995 review (Desiraju, 1995 ▸) made no attempt to consider ‘homosynthons’ and ‘heterosynthons’ as being different types of supramolecular entities.

A great deal of effort has gone into the development of pharmaceutical cocrystals that display better bioavailability when administered. This has resulted in around a half dozen marketed drugs that are based on crystal-engineered logic-driven retrosynthetic strategies. Some of my own work has attempted to find a ‘sweet spot’ in the solubility–permeability dichotomy (Sanphui *et al.*, 2013 ▸; Gopi *et al.*, 2016 ▸). Can one figure out a coformer functionality that, in a small range of structural space, optimizes both solubility and permeability (Maity *et al.*, 2020 ▸)? Could we use AI to fine-tune such a sweet spot if it exists (Desiraju & Bhattacharya, 2025 ▸)? These are questions for the future.

## 6. Polymorphism

The most enigmatic phenomenon I have come across in all my journeys across the domains of crystal engineering is what may loosely be termed ‘polymorphism’, or the occurrence of isolable crystal forms that are different from one another and yet are derived from the same chemical substances in molecular terms. Polymorphism occurs when the same molecular structure leads to different crystal structures (Threlfall, 1995 ▸).

Far from the simple matter that it seems to be, polymorphism is actually an extremely complex issue. The complexity begins with the very definition of the terms ‘molecular structure’ and ‘crystal structure’. To be more specific, a molecular structure may be a function of temperature and pressure, while a crystal structure is both a time-averaged and space-averaged description which is dependent on the methods and power of the resolutions of the various instruments used to determine them (Sarma & Desiraju, 1999 ▸). With reference to the above definition of polymorphism, what is ‘same’ and what ‘different’?

**Figure 11:**
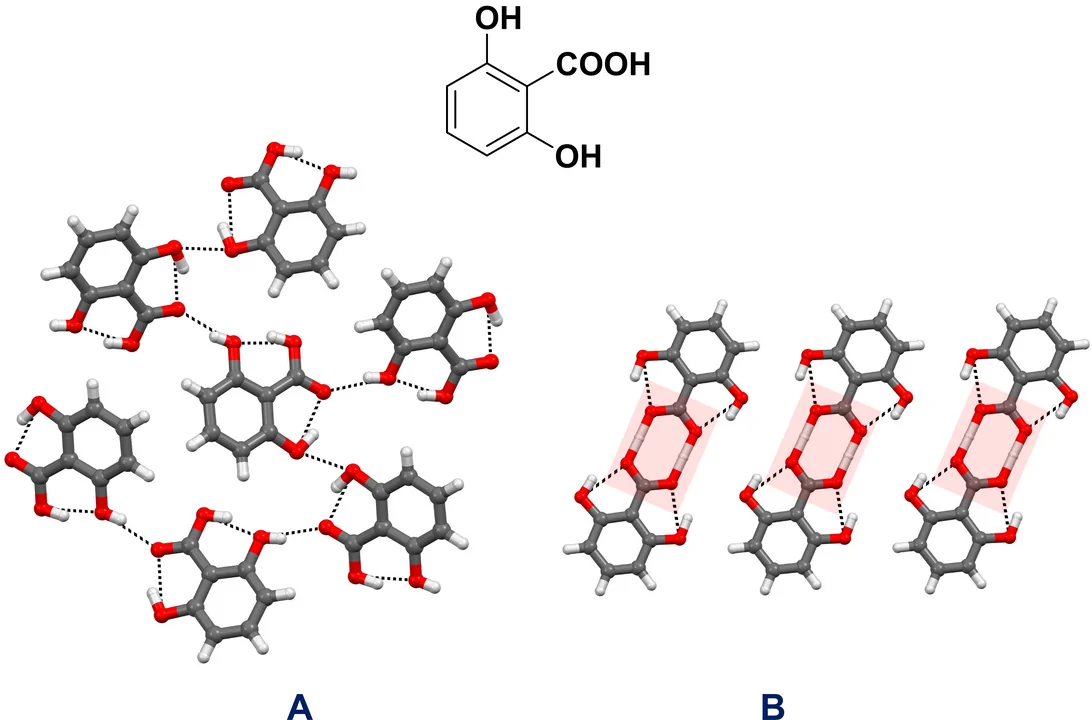
Synthon polymorphism in 2,6-di­hydroxy­benzoic acid.

There are polymorphs where the principal synthons, dare I employ the term ‘structure determining interactions’, a popular descriptor in the past when synthon theory was gaining in acceptance, are the same in the two or more forms. In these cases, the differences in the forms occur mostly in ‘higher’ or ‘larger’ synthons, the so-called Large Synthon Aufbau Modules (LSAM), that define more subtle structural features such as the spatial relationships between ‘lower’ patterns like tapes, ribbons and sheets (Ganguly & Desiraju, 2010 ▸). There are other polymorphs in which the major or, shall we say, principal synthons, typically hydrogen bonded, are in themselves different (Mukherjee & Desiraju, 2011 ▸). In these cases, the molecules are typically multifunctional (Fig. 11 ▸) or, as in the cases where hydrogen-bonding groups such as C—O—H are present, have a certain degree of conformational flexibility, so that topological variation of the main O—H⋯O—H⋯O—H arrangement is seen.

**Figure 12:**
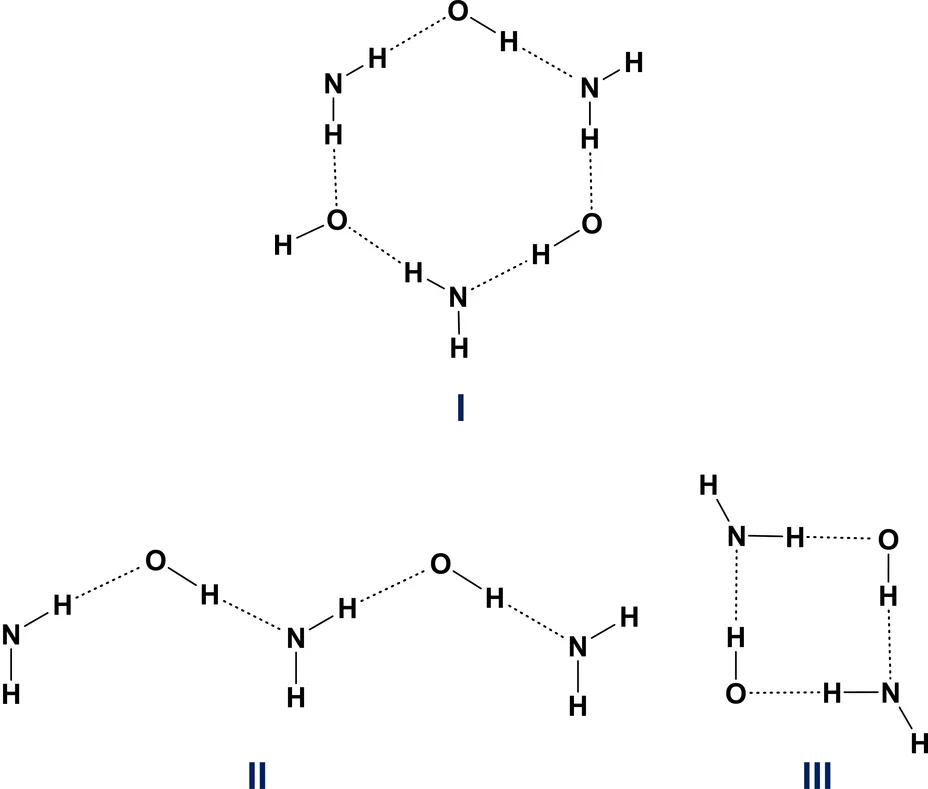
Amino­phenol synthons.

Fascinating variations of such themes can be seen in the amino­phenols, compounds in which the hydrogen-bond donor and acceptor capabilities of the OH and NH_2_ groups are complementary (Fig. 12 ▸). A particular amino­phenol may take one particular crystal structure without necessarily being polymorphic (Allen *et al.*, 1997 ▸; Vangala *et al.*, 2003 ▸; Dey *et al.*, 2005 ▸). This raises the question as to whether one could call a phenomenon ‘polymorphism’ if a group of molecular structures could be taken together as being the ‘molecular structure’ and the group of crystal structures that arise from this ‘molecular structure’ may be taken as ‘the crystal structure’. This leads to the idea that the ‘structure’ itself does not represent anything unique other than being a data point in what may be termed a ‘structural space’. These are new ideas that need to be explored because they offer scope to expand the synthetic exercise into new, more useful functional solids.

Chemistry is all about structure, synthesis and dynamics. If structure itself is not considered a simple attribute but rather is treated as a ‘complex system’, one enters research areas where one may access the third limb of crystal engineering, namely property, the two others being interaction and design. Going further, in the same way that a molecule may have many crystal structures, and a crystal structure, especially if defined topologically, may be associated with many molecules, one may conceive of the same structure being associated with many properties, just as the same property may be associated with many structures. A one-to-one correlation between structure and property has been a hallmark of an older reductionist approach to chemistry, especially the traditional physical organic chemistry of the 1950s and 1960s. I conceived of crystal engineering as a newer type of physical organic chemistry, where one is comfortable with complexity and even welcomes it as an asset that enables one to solve complex problems. Never can this be more apparent than in the quest to find cures for complex diseases like cancer, diabetes and arteriosclerosis, which need to be handled in ways totally different from simple diseases like cholera, typhoid, leishmania and smallpox (Blundell, 2017 ▸). For example, a drug designed to treat protein–ligand recognition as a complex phenomenon may well prove efficacious in treating two seemingly unrelated diseases. Pharmaceutical cocrystals offer a vital link in such treatments and are currently defined by the US FDA as ‘engineered solid-state forms of an API that are composed of the API and one or more coformers in a defined stoichiometric ratio within the same crystal’.

Polymorphism is closely related to the subject of crystal structure prediction (CSP) (Thakur *et al.*, 2015 ▸), which gained popularity in the early 1990s after semi-empirical computations began to be used to calculate the energies of polymorphs of any given compound, in attempts to determine which particular polymorph would be expected to occur under a given set of ambient conditions, namely laboratory synthesis. Following the rationalization of the crystal structure of a particular polymorph, the direction of research shifted to the prediction of new structures (computer synthesis).

In the early so-called ‘blind tests’ for CSP, participants were supplied with a molecular structure in terms of a structural formula and asked to come up with the ‘predicted’ crystal structure, which had already been determined by the persons who had organized the test and held in confidence until all the participants (contestants) had come up with their ‘answers’ based on computations that relied on atom potentials of various types (Lommerse *et al.*, 2000 ▸).

I had decided early on that this simplistic (reductionist) approach to CSP was inadequate and, taking from the idea that the supramolecular synthon is a kinetically stabilized structural fragment (Sarma & Desiraju, 2002 ▸), I used the Cambridge Structural Database (CSD) to identify likely synthons in the crystal structure of the test molecule. This is in accordance with the idea that a given ‘system of molecules’ will give rise to a predicted ‘system of crystal structures’. Accordingly, I provided CSP predictions in the second and subsequent blind tests that relied on synthon-based methodologies. The most likely crystal structures are those that contain the most ‘robust’ synthons. The synthon is finally a probabilistic event and is based on a hierarchy of intermolecular interactions of varying strengths and distance dependence properties. It is a complex convolution of events and interactions. The ‘best’, or those that are most easily identified on the basis of energy–density plots, are the ones most likely to occur. A recent study of marketed APIs, where in-house crystallization protocols have most likely been heavily established, shows that in most cases the marketed forms are the ones that are generally obtained in ‘standard’ protocols (Chappa *et al.*, 2025 ▸) such as would be apparent to persons of ordinary skill in the art (POSA), to use legal terminology. These terminologies are of relevance because different polymorphs may have different criteria to qualify for patents: are they novel, are they useful in new ways and above all, are they non-obvious to a POSA, a strange character who possesses an almost infinite amount of scientific information but has no reasoning ability apart from completely elementary, instinctive, motor skills, a kind of robot.

Things moved on for me in the past decade (Chakraborty *et al.*, 2018 ▸) when I felt that my ‘systems of crystal structures’ explored earlier properly constituted what was being increasingly referred to in the literature as a ‘crystal structure landscape’ (Kirchner *et al.*, 2004 ▸). I made the distinction between ‘a crystal structure’, which is merely a data point in an energy–density hyperspace (reductionist crystal), and ‘the crystal structure’, which is, in the limit, the entire landscape (holistic crystal). Taking this thought stream forward, I can predict that the AI method will solve the problem of crystal structure prediction completely in, say, five to ten years from now, maybe sooner, and even for molecules with increased degrees of flexibility. Diversions like ‘disappearing’ polymorphs or something useful that can only be obtained by crystallizing it from a 1:1:1 mixture of ether, *iso*-octane and neo­pentane have already vanished from the active thinking of the crystal engineer. From a situation where I described polymorphism as the Nemesis of crystal engineering, in my 1989 book, the phenomenon has now become the handmaiden of the subject, the Themis as it were, representing the benevolent and constructive side of the divine order, and one who does not play dice with the universe (Chappa *et al.*, 2025 ▸).

## 7. Mechanically deformable crystals

What is a crystal (Desiraju, 2003 ▸; Bacchi *et al.*, 2014 ▸)? Is it a three-dimensional array of molecules that predictably gives a diffraction pattern when it encounters a beam of X-rays, electrons or neutrons of the appropriate wavelength? Could one include crystals with aperiodic arrays that still give a diffraction pattern? Is one restricted to three-dimensional periodicity? What about solids with lower degrees of order? Are there intermediates in the solution → crystal continuum that might provide routes to understanding the mechanism of crystallization (Mukherjee, Dixit *et al.*, 2014 ▸), the ultimate goal of crystal engineering? We search for a general theory that takes chemical dynamics towards crystal statics.

Diamonds were recognized in India more than 2000 years ago as thunderbolts of the gods (Ray, 1902 ▸); our ancients knew they were hard and abrasive and, as such, could be used to polish softer materials like corundum, topaz and quartz. However, there are many crystals that are actually quite soft and prone to breakage and fracture. Structural investigation of such materials using diffraction-based methods after Laue, Bragg and Ewald (Authier, 2013 ▸) came on the scene were scanty. Such studies were not routine until the mid-1980s. Additionally, there is always the human tendency for a crystallographer to pick the best-looking crystal in a batch, and these do not generally include the above-mentioned soft, brittle or deformable ones. It was around this time (late 1980s) that I had an opportunity to look at crystals of the 4-halogen­ated benzo­nitriles (Desiraju & Harlow, 1989 ▸), with strange shapes and unusually prone to deformation. Studies on the polymorphs of aspirin (Bond *et al.*, 2007*a* ▸,*b* ▸; Varughese *et al.*, 2011 ▸), omeprazole (Bhatt & Desiraju, 2007 ▸; Mishra *et al.*, 2015 ▸) and a series of α,ω-alkane di­carb­oxy­lic acids HO_2_C—(CH_2_)_*n*_—CO_2_H (Mishra *et al.*, 2013 ▸) showed that the softness or brittleness of a crystal could often be rationalized on the basis of anisotropy of interactions within the structure. Crystals where this anisotropy was pronounced showed a greater propensity to shear, bend, or crack when subjected to mechanical deformation. Over the years, crystals have generally been made when they fulfilled a purpose. And so it was with soft, deformable crystals. This has been especially true in the modern context of crystal engineering.

**Figure 13:**
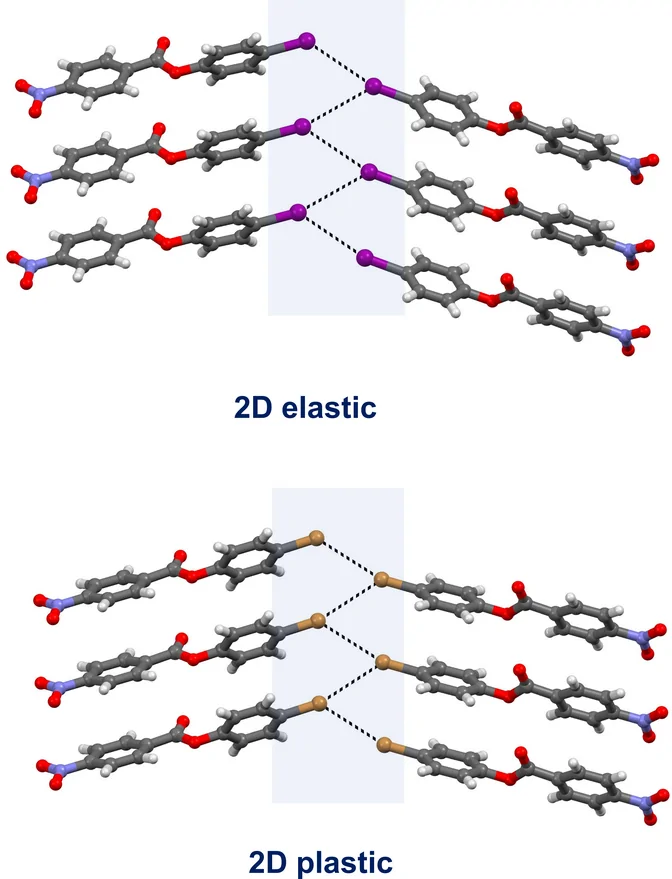
The ‘same’ structure can have different properties

Thus began a new sub-branch of crystal engineering (Saha & Desiraju, 2017*b* ▸), one that employs crystal complexity to design crystals in which one can deliberately and reversibly transition a system from one property to another. We found an interesting case where interactions like Br⋯O and Cl⋯O, which, while occurring in isomorphous structures, have energies that are sufficiently different so that the differing degrees of anisotropy in the crystal structures in which they occur have different mechanical properties – the same structure has different properties (Fig. 13 ▸) (Saha & Desiraju, 2017*a* ▸).

This leads to a final philosophical point. Is matter better defined by structure (earth, water, fire, wind, ether) or by property (smell, touch, sight, feel, sense)? This question is a fundamental one with no answer as long as one confines oneself to simple systems and reductionist thinking, as is the case with nearly all of crystallography as it is practised today and in large segments of chemistry where the structure → property one-to-one correspondence is too deeply ingrained in the mind of the scientist (Ghosh *et al.*, 2015 ▸).

Crystal Engineering grew as an offspring of the parent discipline of supramolecular chemistry in the 1990s. By say 2010, there were a sufficient number of outcrops of Crystal Engineering (Howard *et al.*, 1999 ▸; Tiekink & Vittal, 2006 ▸); I have mentioned the cases of pharmaceutical cocrystals and mechanically deformable crystals that warrant their identification as maturing sub-disciplines. The subject of Crystal Engineering already had two journals, *Crystal Growth & Design* from the American Chemical Society and *Cryst­EngComm* from the Royal Society of Chemistry. The time was ripe for the first Gordon Research Conference on Crystal Engineering in 2010 in New Hampshire. The appearance of this journal, *IUCrJ*, in 2014 as part of the commemoration of the International Year of Crystallography (IYCr) with Chemistry and Crystal Engineering designated as a section, along with Biology and Medicine, CryoEM, Electron Crystallography, Materials and Computation, Neutron and Synchrotron Science and Technology, and finally Physics and Free Electron Laser Science and Technology, has only confirmed what structural chemists had known for some time – that crystal engineering is part of the crystallographic mainstream. We also know since the time I put forth the idea of the supramolecular synthon that crystal engineering is part of the chemistry mainstream. So what is Crystal Engineering? If it is neither chemistry or crystallography and yet also both of these subjects, one is tempted to quote Anderson, who said that the whole becomes not only more than but very different from the sum of its parts (Anderson, 1972 ▸). I would like to affirm, in this context, that crystal engineering is a new subject just as chemistry and crystallography were new subjects when they emerged from older disciplines, chemistry in the early 1800s and (structural) crystallography in the early 1900s.

## 8. Holism

The paradigm of modern science is undergoing a quiet but profound shift. For centuries, we have thrived on the reductionist approach, that one can understand a complex whole by breaking it down to its simplest constituent parts. This approach has given us the wonders of the industrial and digital ages. However, as we confront the layered mysteries of living organisms and advanced materials, the limitations of pure reductionism have become apparent. Increasingly, the global scientific community is turning towards the holistic and integrative philosophies of older societies. This convergence is most visible in the study of complexity, specifically within systems biology, supramolecular chemistry and crystal engineering.

In the crystal engineering context, the supramolecular synthon is the archetype of complexity, and in its design, which involves both strong and weak synthons, one implicitly employs holistic thinking, such that design strategy and methodology become holistically integrated with property and dynamics. This trend has been apparent even in my early work, dating back to the 1990s, even though some of the simpler examples might well have been understood by a reductionist model. The previously mentioned example of CBr_4_ replacing urotropin with a similar shape and size is a case in point. This is an ordered ternary cocrystal. But such a cocrystal cannot be readily imagined as a higher quaternary cocrystal using ‘conventional’ methods. The synthon model is holistic and non-reductionist from its very outset (Desiraju, 2007 ▸).

At the heart of this bridge between scientific systems lies a simple, versatile interaction: the hydrogen bond, often called the interaction of life. The hydrogen bond is not a monolithic force. It exists in a spectrum of strengths, and this duality perfectly mirrors the necessary synergy between scientific systems that coexist rather than mutually contradict.

In its strong manifestation, the hydrogen bond is a pillar of stability. It is predictable, robust and conservative. These hallmarks represent the desire for certainty, the establishment of rigid laws and the focus on structural integrity. We see this in the primary crystal structures of molecules as represented in the more than one million entries in the CSD. These strong depictions of a structure seemingly maintain its form and function over time. They provide the essential blueprint of ‘the crystal structure’.

However, life itself cannot exist on strength alone. To move, to breathe, and to think, a system must be fluid. This is where weaker manifestations of the hydrogen bond – specifically the C—H⋯O and C—H⋯N interactions – become critical. These weak bonds represent adaptability, subtlety and complexity. If strong bonds are the laws of the system, weak bonds are its sociological impulses. They allow molecules to recognize one another and most importantly to self-heal in crystals.

A functional system, such as we try to make in crystal engineering, is a perfect positive synergy between these two forces. It requires structural rigidity of the strong bonds and the adaptive complexity of weak bonds. When this is balanced, the system flourishes. By valuing the weak hydrogen bond as much as the strong one and by integrating the precision of reductionism with the adaptability of holism, we move towards a more sophisticated science that respects the true nature of matter.

Holistic, as opposed to reductionist, science supports the view that the fundamental structure of the human mind is uniform across all races. Our varied cultures are but dialects of a single speech. The differences are due to accents, historical circumstances and stages of development. If we are to find a solution for the differences which divide nations today, it must be through the recognition of the essential oneness of the modern world, socially, economically and politically – and yes, even spiritually. One does not need to believe in God to understand this.

## 9. Revolutions

**Figure 14:**
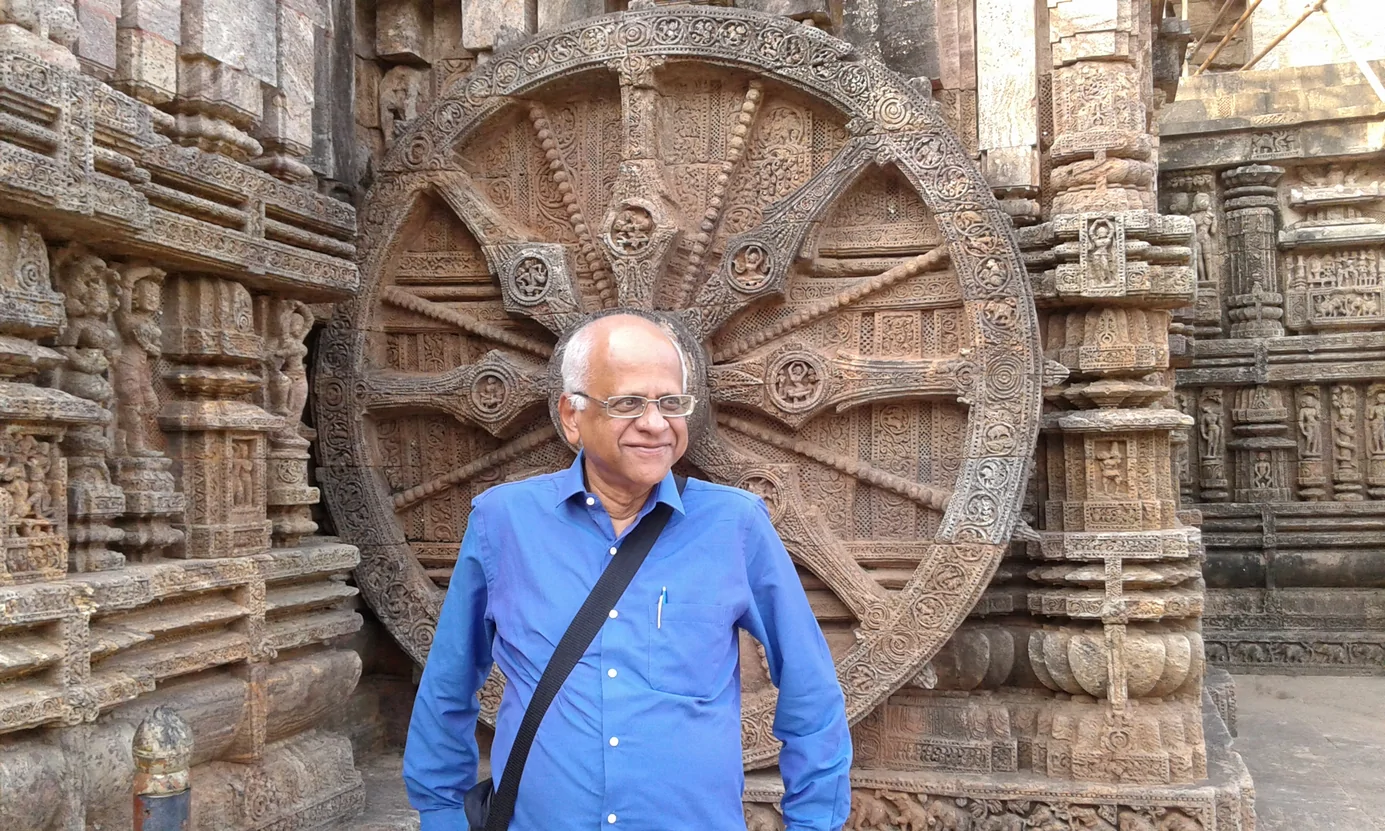
The author at the iconic temple in Konark, Odisha, standing beside one of the sixteen wheels of the chariot of the Sun God, Surya, the source of all the world’s energy.

The past 125 years have witnessed revolutionary changes that stand unparalleled in human history. Scientific inventions have eliminated human isolation and created remarkable opportunities for realizing the long-held aspiration of establishing a cohesive global society. This vision has inspired thinkers and foresighted individuals across various nations. The social and ethical questions arising from the proliferation of science and technology, as well as the newfound interactions among diverse races and cultures, are pertinent to both Eastern and Western societies (Radhakrishnan, 1939 ▸). It is imperative that we cultivate the ability to coexist and foster mutual understanding (Desiraju, 2019 ▸). Unlike what Kipling said, East and West can coexist and indeed they must in the New World Order (Fig. 14 ▸).

Revolutions in the political world are always disruptionist (Durant, 1930 ▸). They are caused by wars or result in wars. They disturb an existing order of things and overcome forces and circumstances that tend to preserve the status quo. Political revolutions are often caused by technological innovation and the inability of such innovations to fully predict the consequences of these innovations – the arrival of the aeroplane in the early 1900s having much to do with the disappearance of four empires after World War I being a typical example. The use of drones in the recent wars in the Ukraine and West Asia is another good example of this phenomenon. Political revolutions are also mostly ‘noisy’ and generally permanent. There is no return to the old order (Strachan, 2013 ▸).

Revolutions, however, occur in areas of human activity that are removed from politics and geostrategy. Scientific revolutions have occurred fairly regularly during the last 500 years, that is to say, in the modern era, and yet these revolutions, although they disrupt and change the existing order of things, are silent when compared with political revolutions. Like political revolutions, or maybe even more so, scientific revolutions can be caused by technological innovations and, similarly, by the inability of these innovations to fully predict their consequences.

In both crystallography and chemistry as practised over the last century, there has been a growing realization that the progress of these subjects, as dramatic and decisive as it has been, has also highlighted the insufficiency, inadequacy and inability of prevailing paradigms of thought to address problems of increasing complexity in the modern world. It is necessary to understand this further. Insufficiency means that existing methods of analysis and research can solve some important problems, but not all of them. Inadequacy means that existing methods can address difficult problems up to a certain point but cannot take them further. Inability means that existing methods cannot even begin to address the problem. My studies in chemical crystallography have led me to encounter all these situations. It has been these experiences that annealed me as a participant into this silent revolution in the structural sciences.

Silent scientific revolutions do not completely overthrow the existing order of things. They simply displace the existing order in selected domains, and over a period of time that may extend to several decades the new order imperceptibly becomes the central order. A new centrality is the key. In this respect, scientific and political revolutions share a common trait – what was on the periphery before now occupies centre stage.

In an article entitled *Crystal Engineering: An Outlook for the Future*, I outlined that crystal engineering leverages the understanding of interactions to design functional solids (Nangia & Desiraju, 2019 ▸). With the increasing impetus to align science with societal needs for practical utility in a world that is becoming even more complex with bewildering speed (Desiraju, 2019 ▸), research and funding will almost certainly prioritize high-impact areas: pharmaceutical solids for improved drug delivery, industrial solid state reactions for efficient synthesis, mechanical properties with direct engineering applications, framework solids for gas storage and catalysis, and new materials for solar energy harvesting and as advanced polymers.

The revolution caused by crystal engineering is a silent one, as are other scientific revolutions of the modern era. In earlier times, scientific revolutions were not necessarily silent or incremental, to wit Lavoisier’s demolition of the phlogiston theory of combustion. The modern disruptions are more subtle and gradual. My wish list for Crystal Engineering of ten items sets out what I feel are important goals over the next decade, by which time the subject would be fully mainstreamed. This list includes: the use of new and conceivable interactions in design strategies; the understanding of microcrystalline solid forms and those that lie outside the domain of order, say solid solutions, eutectics and amorphicity (Janssen *et al.*, 2018 ▸; David, 2002 ▸); the monitoring of molecules’ motion in solids under stress; to be able to fix an upper limit of components in a stoichiometric multicomponent crystal; to fully modulate the property of any cocrystal with respect to its molecular components; to be able to determine the number of polymorphs and pseudopolymorphs that may be realized experimentally under reasonable laboratory conditions, in other words a general solution to the problem of CSP possibly using AI; to establish if there is any connection between *Z*′, the number of molecules in the crystal asymmetric unit, and crystallization mechanisms; and to establish a complete connection between property engineering and crystal engineering, whatever the chemical nature of the compounds and systems being studied.

My 1989 definition of crystal engineering mentioned intermolecular interactions, design strategies and crystal properties. The subject has evolved over three stages. In the first stage, the understanding of existing crystal structures in terms of the interactions they contained was largely accomplished. In the second stage, the design strategy was outlined and accomplished in terms of retrosynthesis with the attendant supramolecular synthons, the very kernels of molecular crystals. In the third stage, what was targeted was a property rather than a structure, in the full realization that there need not be a unique one-to-one correspondence between structure and property (Desiraju *et al.*, 2011 ▸). I am pleased that I have been able to contribute appropriately to each of these three stages as the world of the molecular crystal moves to realms of increasing complexity.
